# Risk factors on healthcare-associated infections among tuberculosis hospitalized patients in China from 2001 to 2020: a systematic review and meta-analysis

**DOI:** 10.1186/s12879-022-07364-9

**Published:** 2022-04-20

**Authors:** Xinliang Liu, Nili Ren, Zheng Feei Ma, Meiling Zhong, Hao Li

**Affiliations:** 1 School of Public Health/Global Health Institute, Wuhan University, No. 115 Donghu Road, Wuhan, 430071 China; 2 Division of Population Health, Health Services Research and Primary Care, School of Health Sciences, Faculty of Biology, Medicine and Health, Manchester Academic Health Sciences Centre, The University of Manchester, Manchester, M13 9PL UK; 3 Wuhan Pulmonary Hospital, Wuhan Institute for Tuberculosis Control, Wuhan, 430030 China; 4 Department of Health and Environmental Sciences, Xi’an Jiaotong-Liverpool University, Suzhou, 215123 Jiangsu China

**Keywords:** Tuberculosis, Healthcare-associated infections, Risk factors, Systematic review and meta-analysis, China

## Abstract

**Background:**

China has been still suffering from high burden attributable to tuberculosis (TB) and healthcare-associated infections (HAIs). TB patients are at high risk to get HAIs. Evidence-based guidelines or regulations to constrain the rising HAIs among TB hospitalized patients are needed in China. The aim of this systematic review and meta-analysis is to investigate the risk factors associated with HAIs among TB hospitalized patients in Chinese hospitals.

**Methods:**

Medline, EMBASE and Chinese Journals Online databases were searched. The search was limited to studies published from January 1st 2001 to December 31st 2020. Meta-analyses of ORs of the risk factors between patients with HAIs and patients without HAIs among TB hospitalized patients were estimated. Heterogeneity among studies was assessed based on the $\widehat{{\uptau }}$^2^ and *I*^2^ statistics to select the meta-analysis model. Review Manager 5.3 was employed and *P* < 0.05 was considered as statistical significance.

**Results:**

851 records were filtered from the databases, of which 11 studies were included in the quantitative meta-analysis. A total of 11,922 TB patients were included in the systematic review and meta-analysis, of which 1133 were diagnosed as having HAIs. Age older than 60 years (OR: 2.89 [2.01–4.15]), complications (OR: 3.28 [2.10–5.13]), diabetes mellitus (OR: 1.63 [1.22–2.19]), invasive procedure (OR: 3.80 [2.25–6.42]), longer than 15 hospitalization days (OR: 2.09 [1.64–2.64]), secondary tuberculosis (OR: 2.25 [1.48–3.42]), smoking (OR: 1.40[1.02–1.93]), underlying disease (OR: 2.66 [1.53–4.62]), and use of antibiotics (OR: 2.77 [2.35–3.27]) were the main risk factors associated with HAIs among TB hospitalized patients with a statistical significance (*P* < 0.05).

**Conclusions:**

Age older than 60 years, presence of complications, presence of diabetes mellitus, invasive procedure, longer than 15 hospitalization days, secondary tuberculosis, smoking, presence of underlying disease, and use of antibiotics were the main risk factors which had a negative impact on HAIs among TB hospitalized patients in Chinese hospitals. These findings provided evidence for policy makers and hospital managers to make effective infection prevention and control measures to constrain the rising HAIs. It is also required that more cost-effective infection prevention and control measures should be widely applied in routinely medical treatment and clinical management to reduce the occurrence of HAIs among TB hospitalized patients.

**Supplementary information:**

The online version contains supplementary material available at 10.1186/s12879-022-07364-9.

## Introduction

Tuberculosis (TB) is a chronic communicable disease. It has been acknowledged as one of the 10 top causes of disability and a leading cause of death globally [1, 2]. The World Health Organization (WHO) reported that there was a significant drop in the number of newly diagnosed TB patients from 7.1 million in 2019 to 5.8 million in 2020, and China has made significant contributions to the reduction [3]. However, the pandemic of COVID-19 has reversed the deduction on TB incidence because of deteriorated Gross Domestic Product (GDP) per capita and undernutrition, which are the two key determinants of TB incidence [4]. Moreover, the COVID-19 pandemic has reduced the provision of essential TB services and TB burden, especially in those countries without a universal health coverage [3]. The WHO estimated that TB cases would increase by more than 1 million per year in the period 2020-2025 because of the COVID-19 pandemic [4], exacerbating the situation of diminishing TB. China has still been faced with a high burden attributable to TB, especially drug resistant TB. For example, 14% of global rifampicin resistant TB cases in 2019 were detected in China, ranking the second after India [5].

Hospitals, as the main body to provide health care to treat TB patients, are also the main repository of healthcare-associated infections (HAIs). HAIs are an adverse outcome during health care provision to patients in hospitals. It affects patients’ safety and extends hospitalization days, thereby resulting in the increase of morbidity and mortality [6, 7]. HAIs are also a global public health problem. The HAIs prevalence in China was estimated around 3 out of every 100 hospitalized patients in 2018 [8]. Although it was lower than the figure reported by the WHO, China has the largest population in the world. This indicates China has a high burden attributable to HAIs.

Usually, a TB patient needs to be treated with multiple types of antibiotics for 6–12 months [6]. This results in a long time exposure to antibiotics for patients. Antibiotics use in many studies has been recognised as a risk factor to increase HAIs incidence [9, 10]. For TB patients, especially those elderly, they have a low immunity to fight against the infections [11]. As a consequence, TB patients are at high risk to get HAIs. Therefore, it is essential for health professionals and managers to understand what kinds of risk factors have an impact on HAIs among TB hospitalized patients. Thus, it can contribute to guideline and regulation about constraining the occurrence of HAIs among TB hospitalized patients in hospitals.

Several Chinese studies investigating the potential risk factors on HAIs among TB hospitalized patients have been found [12, 13]. However, these studies were based on a single hospital. This calls for evidence-based guidelines or regulations based on the national data to reduce the rising HAIs among TB hospitalized patients. Moreover, it is necessary to help health professionals and hospital managers to increase the awareness of the hazards of HAIs among TB hospitalized patients and take effective infection control measures to constrain the occurrence of HAIs among TB hospitalized patients. Therefore, the aim of this systematic review and meta-analysis is to investigate the risk factors associated with HAIs among TB hospitalized patients by comparing the odds ratio (OR) of risk factors between patients with HAIs and those without HAIs in Chinese hospitals.

## Methodology

### Systematic search strategy

The PICO/S (Population, Intervention, Comparison, Outcome, and Study type) tool was applied to define the scope of the literature.

Population: TB hospitalized patients admitted to hospitals more than 48 hours.

Intervention: HAIs diagnosed as the infections acquired by the patients admitted to a healthcare facility more than 48 h according to the definition proposed by the Ministry of Health, People’s Republic of China [14].

Comparison: TB hospitalized patients without HAIs.

Outcome: HAIs prevalence.

Study type: cross-sectional study, case-control study or cohort study.

This systematic review was conducted in compliance with the Preferred Reporting Items for Systematic Reviews and Meta-analysis (PRISMA) guidelines. Medline, EMBASE and Chinese Journals Online databases (China National Knowledge Infrastructure [CNKI], Chinese Wan Fang digital database and Chinese Science and Technique Journals Database [VIP]) were searched. These studies published were limited from 1st January 2001 to31st December 2020, since the version of HAIs diagnosis was first published in 2001 in China.

As to Medline and EMBASE, medical subject heading (MeSH) terms in key words were adopted to search the studies published in English. The MeSH terms were (“cross infection” AND “tuberculosis” AND “risk factors” AND “China”). Chinese corresponding terms in the title, abstract and keywords were searched including healthcare-associated infections/cross infections/hospital acquired infections/nosocomial infections, tuberculosis, risk factors/influencing factors/ and China for the Chinese databases. The logical word is “AND”.

### Inclusion and exclusion criteria

The inclusion criteria included: (1) risk factors analysis by using a cross-sectional, case-control or cohort study; (2) a multi-centre study or a single-centre study; (3) study language being either English or Chinese.

The exclusion criteria included: (1) conference papers or editorials/letters; (2) duplicate studies and repeated data published in different journals concurrently; (3) any study outside China; (4) only description on the prevalence or landscape of HAIs; (5) risk factors concluded without any statistical inference (e.g. according to doctors' clinical experience).

### Data abstraction

Two independent reviewers (XL and NR) screened the obtained literature by title and abstract to determine the eligibility of studies. Potential disagreement was resolved by discussion. Identified studies were retrieved in full text and were checked again for eligibility. In case of exclusion of the study, the reason was documented. Moreover, cross-references were also considered by screening the bibliography of eligible studies as well as bibliography of cross-references.

### Quality assessment of the included studies

XL and NR jointly assessed the quality of the included studies for risk factors analysis associated with HAIs among TB hospitalized patients according to the criteria of JBI’ (Joanna Briggs Institute) critical appraisal tools [15]. This tool includes 8 questions to identify the quality of a cross-sectional study. For each question, there are 4 options to choose (Yes, Unclear, No and Not applicable). The less the number of a positive option (Yes) is, the more the uncertainty of a study is. Otherwise, the quality of a study is better. Moreover, a value was assigned to each answer in order to calculate the scores for each study, that is, 2 points for ‘Yes’, 1 point for ‘Unclear or Not applicable’, and 0 point for ‘No’.

### Statistical analysis

Review Manager 5.3 software was deployed to assess the risk factor odds ratio (OR) among TB hospitalized patients. Meta-analyses of ORs of the risk factors between hospitalized patients with HAIs and hospitalized patients without HAIs were performed. Heterogeneity between studies was assessed based on the $\widehat{{\uptau }}$^2^ and *I*^2^ statistics to select the meta-analysis model. When results had a $\widehat{{\uptau }}$^2^ (*P* < 0.05) and/or *I*^2^ > 50%, data were considered heterogeneous and the random-effects model was used; otherwise, the fixed-effects model was used. *P* < 0.05 was considered as a statistical significance.

## Results

### Characteristics of eligible studies

**Fig. 1:**
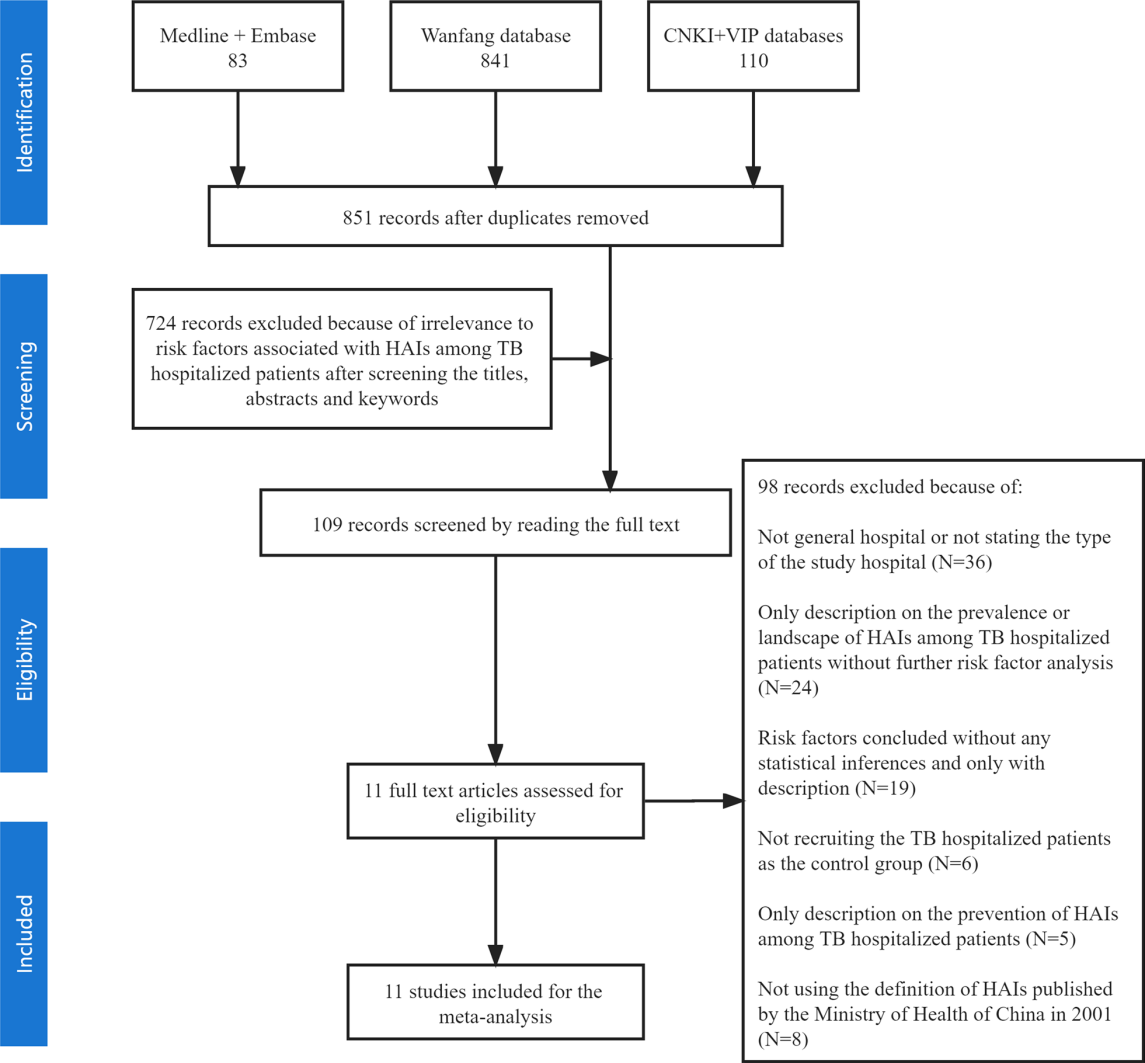
Flow chart of the searching relevant published articles from the included databases. *CNKI* China National Knowledge Infrastructure, *Wangfang database* Chinese Wan Fang digital database, *VIP* Chinese Science and Technique Journals Database, *HAIs* Healthcare-associated infections, *TB* tuberculosis

Figure 1 shows that totally, 851 records were searched from the included databases. Eventually, 11 published articles were incorporated in the quantitative meta-analysis after reading the eligible full texts according to the inclusion and exclusion criteria.

### Integration of the included studies on risk factors between patients with HAIs and patients without HAIs among TB hospitalized patients

Table 1 presents that in total, 11,922 TB hospitalized patients were included in the systematic review and meta-analysis. Of them, 1133 were diagnosed as having HAIs, while the rest (11,789) were not with HAIs. All of the included studies were undertaken in tertiary general hospitals, which covered 10 regions in 9 provinces in China. Table 2 shows that the most frequent risk factors reported from the included studies were age (11/11), use of antibiotics (11/11), invasive procedure (9/11), and length of hospitalization (9/11).

**Table 1:** Characteristics of the studies included in the systematic and meta-analysis on risk factors for healthcare-associated infections among TB hospitalized patients in Chinese hospitals from 2001 to 2020

Study ID	Author (year)	Study design	Study setting	Region (province)	Year begun and duration (years)	Number of participants	
						HAIs	Non-HAIs
1	Chen et al. (2018) [16]	Retrospective cross-sectional	One tertiary hospital	Wenling (Zhejiang)	2014, 3	28	653
2	Dai et al. (2014) [17]	Retrospective cross-sectional	One tertiary hospital	Wenzhou (Zhejiang)	2012, 1	30	286
3	Liu et al. (2015) [18]	Retrospective cross-sectional	One tertiary hospital	Zhuhai (Guangdong)	2005, 9	79	1225
4	Zeng (2015) [19]	Retrospective cross-sectional	One tertiary hospital	Foshan (Guangdong)	2010, 4	19	223
5	Zhang et al. (2012) [20]	Retrospective cross-sectional	One tertiary hospital	Weihui (Henan)	2008, 3.5	117	756
6	Zhong et al. (2010) [21]	Retrospective cross-sectional	One tertiary hospital	Foshan (Guangdong)	2007, 1	56	1306
7	Zhou et al. (2018) [22]	Retrospective cross-sectional	One tertiary hospital	Baoding (Hebei)	2015, 2.5	152	2234
8	Huang et al. (2019) [12]	Retrospective cross-sectional	One tertiary hospital	Danzhou (Hainan)	2015, 1	78	78
9	Jiang et al. (2019) [23]	Retrospective cross-sectional	One tertiary hospital	Xining (Qinghai)	2016, 1.5	32	269
10	Xiang et al. (2019) [13]	Retrospective cross-sectional	One tertiary hospital	Qiongqing	2015, 2	400	1901
11	Gong & Wang (2013) [41]	Retrospective cross-sectional	One tertiary hospital	Binzhou (Shandong)	2006, 7	142	1858

**Table 2:** Frequency of the identified risk factors in the included studies on risk factors for healthcare-associated infections among TB hospitalized patients in Chinese hospitals from 2001 to 2020

Study ID/Risk factors	1	2	3	4	5	6	7	8	9	10	11	Total
Age	√	√	√	√	√	√	√	√	√	√	√	**11**
AIDS						√						**1**
Albumin (> 40 g/L vs. ≤ 40 g/L)		√										**1**
Complications					√					√	√	**3**
Course of disease (> 5 years vs. ≤ 5 years)		√										**1**
Diabetes mellitus			√			√						**2**
Gender	√	√	√	√	√	√	√		√			**8**
Invasive procedure		√	√	√	√		√	√	√	√	√	**9**
Length of hospitalization		√	√	√	√		√	√	√	√	√	**9**
Negativity of the sputum smear for acid fact staining	√	√										**2**
Secondary tuberculosis	√	√				√						**3**
Smoking			√		√							**2**
Tuberculosis retreatment	√											**1**
Underlying diseases	√	√	√	√				√	√			**6**
Use of antibiotics	√	√	√	√	√	√	√	√	√	√	√	**11**
Use of anti-tuberculous drug				√				√				**2**
Use of glucocorticoid	√											**1**

Secondary tuberculosis is defined that patients are re-infected with the tubercle bacillus or re-activated from a dormant endogenous infection [42]. Complications are defined that TB patients are concurrently diagnosed as having other kind of disease

### Quality assessment of the included studies

All of the 11 included studies were cross-sectional with a median score (10 points). All of the studies clearly defined the inclusion for the hospitalized patients, identified the confounding factors which had an impact on the occurrence of HAIs among TB hospitalized patients, and measured the outcomes in a valid and reliable way. However, none of the studies described the study setting with more details and only mentioned the hospital without any details. Also, variation of the HAIs prevalence existed among the included studies. It mainly showed that some studies did not describe how the HAIs prevalence among the TB hospitalized patients was calculated. The same situation was also applied to the standard criteria for the TB hospitalized patients with HAIs and the strategy to deal with the confounding factors (see Additional file 1: Appendix 1)

### Meta-analyses of all the potential risk factor ORs

Table 3 shows that a total of 11 risk factors were included in the meta-analyses between TB hospitalized patients with HAIs and TB hospitalized patients without HAIs. The forest plot of each risk factor was presented in Additional file 1: Appendix 2. Specific results were shown as follows.

**Table 3:** The pooled ORs of the risk factors estimated in the meta-analyses between the patients with HAIs and the patients without HAIs among TB hospitalized patients

Risk factors	Number of patients		Meta-analysis model	OR [95% CI]	^2^(*P*)	*Ι*^2^ (%)	*P-*value
	HAIs	Non-HAIs					
Age (≥ 60 years vs. <60 years)	481	6686	Random-effects	2.89 [2.01–4.15]	0.14 (0.006)	67	< 0.01*
Complications	103	2182	Fixed-effects	3.28 [2.10–5.13]	–	0	< 0.01*
Diabetes mellitus	231	3459	Fixed-effects	1.63 [1.22–2.19]	–	0	0.0009*
Gender	916	7402	Fixed-effects	1.04 [0.90–1.21]	–	0	0.55
Invasive procedure	512	5805	Random-effects	3.80 [2.25–6.42]	0.39 (< 0.01)	78	< 0.01*
Length of hospitalization (> 15 days vs. ≤ 15 days)	317	4971	Fixed-effects	2.09 [1.64–2.64]	–	44	< 0.01*
Negativity of the acid-fast bacilli (AFB) smear and culture	432	2170	Random-effects	1.23 [0.41–3.65]	0.54 (0.006)	87	0.71
Secondary tuberculosis	511	3,395	Random-effects	2.25 [1.48–3.42]	0.08 (0.10)	56	0.0001*
Smoking	180	2887	Fixed-effects	1.40 [1.02–1.93]	–	0	0.04*
Underlying disease	779	5238	Random-effects	2.66 [1.53–4.62]	0.33 (< 0.01)	87	0.0005*
Use of antibiotics	991	8931	Fixed-effects	2.77 [2.35–3.27]	–	45	< 0.01*

*means statistical significance at *P *< 0.05

Age (> 60 years vs. ≤ 60 years): 7 studies [16–22] reported that 279 patients with and 2327 patients without HAIs were over 60 years old. Random-effects model showed that the prevalence of HAIs was significantly higher (*P* < 0.01) in patients who were over 60 years old than those who were younger than 60 years old (OR: 2.89, 95% CI: 2.01–4.15). There was a middle level of heterogeneity among the studies ($\widehat{{\uptau }}$^2^=0.14 (*P* = 0.006), I^2^ = 67%).

Complications: 3 studies [16, 19, 21] reported that 74 patients with and 943 patients without HAIs had the complications. Fixed-effects model showed that the prevalence of HAIs was significantly higher (*P* < 0.01) in patients with complications than those without complications (OR: 3.28, 95% CI: 2.10–5.13). The heterogeneity did not exist among the studies (*P* = 0.74, I^2^ = 0%).

Diabetes mellitus: 2 studies [18, 22] reported that 103 patients with and 1177 patients without HAIs had the diabetes mellitus. Fixed-effects model showed that the prevalence of HAIs was significantly higher (*P* = 0.0009) in patients with diabetes mellitus than those without diabetes mellitus (OR: 1.63, 95% CI: 1.22–2.19). The heterogeneity did not exist among the studies (*P* = 0.75, I^2^ = 0%).

Gender: 8 studies [12, 13, 16–18, 20, 22, 23] reported 539 patients with and 4,331 patients without HAIs were male. Fixed-effects model showed that the prevalence of HAIs was almost equal (*P* = 0.55) between male patients and female patients (OR: 1.04, 95% CI: 0.90–1.21). The heterogeneity did not exist among the studies (*P* = 0.83, I^2^ = 0%).

Invasive procedure: 8 studies [12, 16, 17, 19–23] reported that 225 patients with and 1830 patients without HAIs experienced the invasive procedure. Random-effects model showed that the prevalence of HAIs was significantly higher (*P* < 0.01) in patients who experienced invasive procedure than those who did not experience invasive procedure (OR: 3.80, 95% CI: 2.25–6.42). There was a high heterogeneity among the studies ($\widehat{{\uptau }}$^2^=0.39 (*P* < 0.01), I^2^ = 78%).

Length of hospitalization (> 15 days vs. ≤15 days): 6 studies [16, 17, 19, 21–23] reported that 200 patients with and 2,318 patients without HAIs stayed in hospital longer than 15 days. Fixed-effects model showed that the prevalence of HAIs was significantly higher (*P* < 0.01) in patients who had length of hospitalization over 15 days than those who had length of hospitalization less than 15 days (OR: 2.09, 95% CI: 1.64–2.64). The heterogeneity did not exist among the studies (*P* = 0.11, I^2^ = 44%).

Negativity of the acid-fast bacilli (AFB) smear and culture: 2 studies [13, 23] reported that 77 patients with and 310 patients without HAIs had the negativity of the AFB smear and culture. Random-effects model showed that the prevalence of HAIs was higher (*P* = 0.71) in patients who had negativity of the acid-fast bacilli (AFB) smear and culture than those who had positivity of the acid-fast bacilli (AFB) smear and culture (OR: 1.23, 95% CI: 0.41–3.65). There was a high heterogeneity among the studies ($\widehat{{\uptau }}$^2^=0.54 (*P* = 0.006), I^2^ = 87%).

Secondary tuberculosis: 3 studies [13, 18, 23] reported that 145 patients with and 611 patients without HAIs were diagnosed with the secondary tuberculosis. Random-effects model showed that the prevalence of HAIs was significantly higher (*P* = 0.0001) in patients who had secondary tuberculosis than those who did not have secondary tuberculosis (OR: 2.25, 95% CI: 1.48–3.42). There was a middle level of heterogeneity among the studies ($\widehat{{\uptau }}$^2^=0.08 (*P* = 0.10), I^2^ = 56%).

Smoking: 2 studies [16, 22] reported that 111 patients with and 1,500 patients without HAIs had the history of smoking. The fixed-effects model showed that the prevalence of HAIs was higher (*P* = 0.04) in patients who smoked than those who did not smoke (OR: 1.40, 95% CI: 1.02–1.93). The heterogeneity did not exist among the studies (*P* = 0.59, I^2^ = 0%).

Underlying disease: 5 studies [12, 13, 20, 22, 23] reported that 322 patients with and 1657 patients without HAIs had the underlying disease. The random-effects model showed that the prevalence of HAIs was significantly higher (*P* = 0.0005) in patients with underlying disease than those without underlying disease (OR: 2.66, 95% CI: 1.53–4.62). There was a high heterogeneity among the studies ($\widehat{{\uptau }}$^2^=0.33 (*P* < 0.01), I^2^ = 87%).

Use of antibiotics: 10 studies [12, 13, 16–23] reported that 561 patients with and 4306 patients without HAIs used the antibiotics. Fixed-effects model showed that the prevalence of HAIs was significantly higher (*P* < 0.01) in patients who used antibiotics than those who did not use antibiotics (OR: 2.77, 95% CI: 2.35–3.27). The heterogeneity did not exist among the studies (*P* = 0.06, I^2^ = 45%).

## Discussion

To our best knowledge, our systematic review and meta-analysis first provided a comprehensive analysis of risk factors on HAIs among TB hospitalized patients in Chinese hospitals. Our review found that age older than 60 years, presence of complications, presence of diabetes mellitus, invasive procedure, longer than 15 days of hospitalization stay, secondary tuberculosis, smoking, presence of underlying disease, and use of antibiotics were the main risk factors which had a negative impact on HAIs among TB hospitalized patients in Chinese hospitals. These findings provided evidence for policy makers and hospital managers to make effective infection prevention and control measures to constrain the rising HAIs.

Our systematic review found that TB hospitalized patients older than 60 years were more susceptible to HAIs than those younger than 60 years (OR: 2.89 [2.01–4.15]). The number of aged population is rapidly rising in the globe. 1 in 6 people beyond the age of 65 years has been estimated in the world in 2050, which is much higher than 1 in 11 in 2019 [24]. Elderly TB patients are recognized as the immune-compromised patients, who are at high risk of acquiring HAIs [25]. Moreover, the elderly TB patients tend to have underlying disease or other comorbidities and need longer time to be treated in hospital, all of which increase the risk of getting HAIs [23]. This suggests that the elderly TB patients should be set as the priority and the surveillance of the elderly TB patients should be strengthened. Thus, it can result in decreasing the risk of getting HAIs among aged TB patients.

Presence of complications, diabetes mellitus, underlying disease, and secondary tuberculosis were found the main factors associated with high HAIs prevalence among TB hospitalized patients in our review. All of them mainly deteriorate the TB patients’ immune systems. Consequently, TB patients were more susceptible to HAIs compared with those without above conditions. One study has confirmed that it was 2.5 times more likely to develop TB among patients with diabetes mellitus in developed countries [26]. It was also found that diabetes mellitus was highly associated with multi-drug resistant TB (MDR-TB) in Asia (OR: 1.40 [1.01–1.95]) in a systematic review and meta-analysis [27, 28]. Moreover, diabetes mellitus is one of the determinants to increase the incidence of TB [3]. Presence of complications, diabetes mellitus, underlying disease, and secondary tuberculosis definitely affect the safety and prognosis of TB hospitalized patients with HAIs patients, and put a challenge to the worldwide health system infection control strategy and clinical management, which further alert the global health development without any control measure.

The hazard of acquiring HAIs among TB hospitalized patients who had invasive procedure was higher than those without invasive procedure (3.80 [2.25–6.42]) in our review. Invasive procedure was actually not frequently reported that had a negative impact on the occurrence of HAIs among TB hospitalized patients in existing research. However, invasive procedure, such as indwelling invasive device and surgery, indeed make the TB hospitalized patients predisposed to HAIs. A single-centre point-prevalence survey in an American hospital showed that 96.8% of hospitalized adult patients had at least one indwelling device [29]. It indicated that invasive procedure was still widely adopted as a treatment among hospitalized patients, which put the patients into high exposure to HAIs.

Our review also found that smoking increased the risk of obtaining HAIs among TB hospitalized patients (OR: 1.40 [1.02–1.93]). Smoking, as a risky behaviour, can compromise the successful TB treatment due to poor adherence to the treatment among TB patients [30]. Additionally, the majority of TB hospitalized patients belong to pulmonary TB and the behaviour of smoking is closely related to the lung disease [31]. This suggests that pulmonary TB hospitalized patients are more likely to acquire healthcare-associated pneumonia if the TB patients smoke. Therefore, TB patients who smoke, especially male patients, need to experience aspiration of sputum, fibrotic bronchoscope, and trachea cannula and so on due to the rising respiratory secretions in lung, thereby increasing the likelihood of HAIs. Personal intervention should be made for TB patients who smoke to stop the risky behaviour. Hence, the treatment of TB could be not more sophisticated and decrease the risk of getting HAIs.

TB hospitalized patients who stayed in hospital longer than 15 days were found to be more likely to obtain HAIs compared with those who had less than 15 days of hospitalization (OR: 2.09[1.64–2.64]) in our review. Prolonged hospitalization stay extends the exposure to HAIs among TB hospitalized patients, which is confirmed in some studies [32, 33]. TB hospitalized patients who stay longer in hospital are more likely to suffer from underlying disease, complications, invasive procedure and so on [34, 35]. As previously mentioned, they promote the probability of the exposure to HAIs among TB hospitalized patients. It is necessary for the hospitals to rationally shorten the hospitalization stay for the TB patients in order to lower the risk of HAIs exposure.

TB hospitalized patients were more risky to get HAIs if they used antibiotics during the treatment compared with their counterparts (OR: 2.77[2.35–3.27]). This finding is consistent with current studies [36]. Use of anti-tuberculosis drug is the main way to treat TB for a long period [23]. During the treatment period, it is common to use antibiotics for TB patients concurrently. As a consequence, an increasing number of TB patients have been infected with multi-drug resistance. Goedele et al. also mentioned that successful TB treatment is compromised by drug resistance because of irrational use of antibiotics to treat TB patients [30]. This indicates that prudent and high-quality antibiotics prescription and rational use of antibiotics are essential to constrain the overuse of antibiotics, thereby reduction in the occurrence of HAIs among TB hospitalized patients. Moreover, antimicrobial stewardship program has been widely recommended as one way to achieve the rational use of antibiotics, including making straightforward and strict rational use of antibiotics guidelines and regulations, improving the clinical doctors’ antibiotics prescription behaviour, and increasing the awareness of antimicrobial resistance among the public [37, 38].

The key to preventing the HAIs among TB hospitalized patients after clearly investigating the risk factors associated with HAIs among TB hospitalized patients is to implement effective infection prevention and control measures. Current literature has suggested that the exposure and transmission of HAIs among TB hospitalized patients can be reduced with the implementation of infection prevention and control guidelines, which can increase the identification and isolation of the potential TB hospitalized patients with HAIs [39]. The United States, WHO, and other institutions have recommended effective infection prevention and control measures to decrease the occurrence of HAIs among TB hospitalized patients, particularly a hierarchy of effective infection control measures, including administrative controls, environmental controls, and personal respiratory protection [40]. Specially, the administrative controls are considered as the first and most important component to decrease the exposure to HAIs among TB hospitalized patients. It is also recommended that more cost-effective infection prevention and control measures be widely applied in routinely medical treatment and clinical management to TB hospitalized patients with HAIs, like hand hygiene. Hence, the exposure and transmission of HAIs among TB hospitalized patients can be reduced and prevented.

Our systematic review and meta-analysis has some limitations. First, the included studies were mostly published in Chinese journals since they were conducted in Chinese general hospitals. It encourages the researchers to publish their findings about risk factors on HAIs among TB hospitalized patients in English journals to share the advanced knowledge. Second, we found the included studies were all conducted in a single hospital. It is required that multi-centre studies could be undertaken in future research to strengthen the current evidence base.

## Conclusions

Age older than 60 years, presence of complication, presence of diabetes mellitus, invasive procedure, longer than 15 days of hospitalization stay, secondary tuberculosis, smoking, presence of underlying disease, and use of antibiotics were the main risk factors which had a negative impact on HAIs among TB hospitalized patients in Chinese hospitals. These findings provided evidence for the policy makers and hospital managers to make effective infection prevention and control measures to constrain the rising HAIs, like a hierarchy of effective infection control measures including administrative controls, environmental controls, and personal respiratory protection or antimicrobial stewardship program. It is also required that more cost-effective infection prevention and control measures should be widely applied in routinely medical treatment and clinical management to HAIs among TB hospitalized patients. Hence, the exposure and transmission of HAIs among TB hospitalized patients can be reduced and prevented.

## Supplementary Information

**Additional** **file 1: Appendix1. **Quality assessment of the included studies with JBI tools. **Appendix 2: **Forest plots of the riskfactors in the meta-analyses.

## Data Availability

The datasets and materials analysed during the current study are available from the corresponding author on reasonable request.
