# Supplementary material for: Risk factors on healthcare-associated infections among tuberculosis hospitalized patients in China from 2001 to 2020: a systematic review and meta-analysis
Source: BMC Infect Dis. 2022 Apr 20;22:392. doi: 10.1186/s12879-022-07364-9 (PMC9019792; doi:10.1186/s12879-022-07364-9)
Supplement: Supplementary file 1 — Additional file 1: Appendix1. Quality assessment of the included studies with JBI tools. Appendix 2: Forest plots of the riskfactors in the meta-analyses. [file 12879_2022_7364_MOESM1_ESM.docx]

**Supplementary material**

**Appendix 1: Quality assessment of the included studies with JBI tools**

**Table 1 Quality assessment of the included studies on risk factors associated with high occurrence of HAIs among TB hospitalized patients with JBI checklist for cross-sectional study**

| Questions/ Study | | Xiang et al. (2019) | Jiang et al. (2019) | Zhou et al. (2018) | Huang et al. (2019) | Chen et al. (2018) | Liu et al. (2015) | Dai et al. (2014) | Gong & Wang (2013) | Zhang et al. (2012) | Zhong et al. (2010) | Zeng (2015) |
| --- | --- | --- | --- | --- | --- | --- | --- | --- | --- | --- | --- | --- |
| 1. Were the criteria for inclusion in the sample clearly defined? | Yes | √ | √ | √ | √ | √ | √ | √ | √ | √ | √ | √ |
|  | Unclear |  |  |  |  |  |  |  |  |  |  |  |
|  | No |  |  |  |  |  |  |  |  |  |  |  |
|  | Not applicable |  |  |  |  |  |  |  |  |  |  |  |
| 2. Were the study subjects and the setting described in detail? | Yes |  |  |  |  |  |  |  |  |  |  |  |
|  | Unclear |  |  |  |  |  |  |  |  |  |  |  |
|  | No | √ | √ | √ | √ | √ | √ | √ | √ | √ | √ | √ |
|  | Not applicable |  |  |  |  |  |  |  |  |  |  |  |
| 3. Was the exposure measured in a valid and reliable way? | Yes |  |  |  |  |  |  |  |  |  |  |  |
|  | Unclear | √ | √ | √ | √ | √ |  |  |  | √ | √ |  |
|  | No |  |  |  |  |  | √ | √ | √ |  |  | √ |
|  | Not applicable |  |  |  |  |  |  |  |  |  |  |  |
| 4. Were objective, standard criteria used for measurement of the condition? | Yes | √ | √ | √ | √ |  |  |  |  |  |  |  |
|  | Unclear |  |  |  |  | √ |  |  |  | √ | √ |  |
|  | No |  |  |  |  |  | √ | √ | √ |  |  | √ |
|  | Not applicable |  |  |  |  |  |  |  |  |  |  |  |
| 5. Were confounding factors identified? | Yes | √ | √ | √ | √ | √ | √ | √ | √ | √ | √ | √ |
|  | Unclear |  |  |  |  |  |  |  |  |  |  |  |
|  | No |  |  |  |  |  |  |  |  |  |  |  |
|  | Not applicable |  |  |  |  |  |  |  |  |  |  |  |
| 6. Were strategies to deal with confounding factors stated? | Yes | √ | √ | √ | √ |  |  |  | √ |  |  |  |
|  | Unclear |  |  |  |  |  |  |  |  |  |  |  |
|  | No |  |  |  |  | √ | √ | √ |  | √ | √ | √ |
|  | Not applicable |  |  |  |  |  |  |  |  |  |  |  |
| 7. Were the outcomes measured in a valid and reliable way? | Yes | √ | √ | √ | √ | √ | √ | √ | √ | √ | √ | √ |
|  | Unclear |  |  |  |  |  |  |  |  |  |  |  |
|  | No |  |  |  |  |  |  |  |  |  |  |  |
|  | Not applicable |  |  |  |  |  |  |  |  |  |  |  |
| 8. Was appropriate statistical analysis used? | Yes | √ | √ | √ | √ | √ |  | √ | √ | √ | √ | √ |
|  | Unclear |  |  |  |  |  |  |  |  |  |  |  |
|  | No |  |  |  |  |  | √ |  |  |  |  |  |
|  | Not applicable |  |  |  |  |  |  |  |  |  |  |  |

**Appendix 2: Forest plots of the risk factors in the meta-analyses**


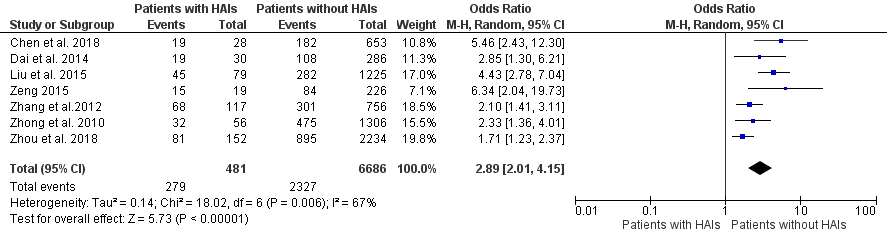
 **
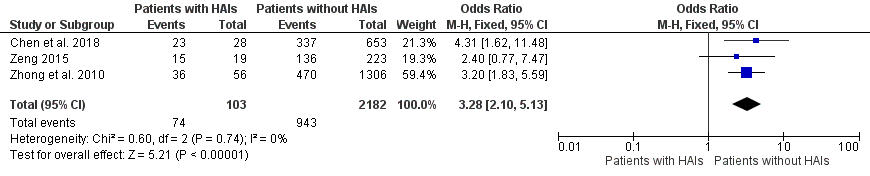

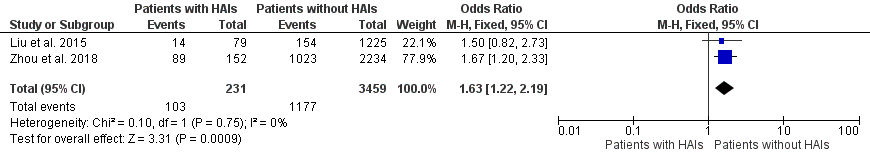
**

**Figure 1-1 Age (≥ 60 years vs. < 60 years) Figure 1-2 Complications (Yes vs. No) Figure 1-3 Diabetes mellitus (Yes vs. No)**

***
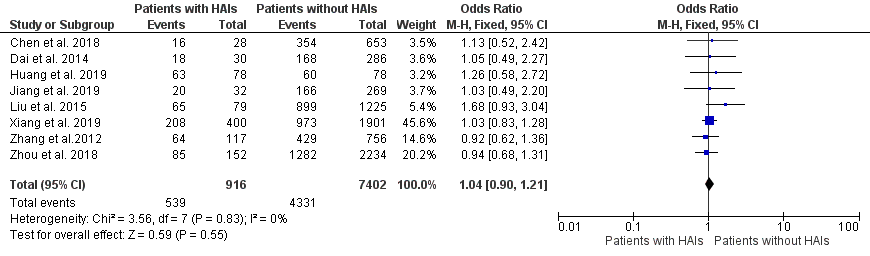

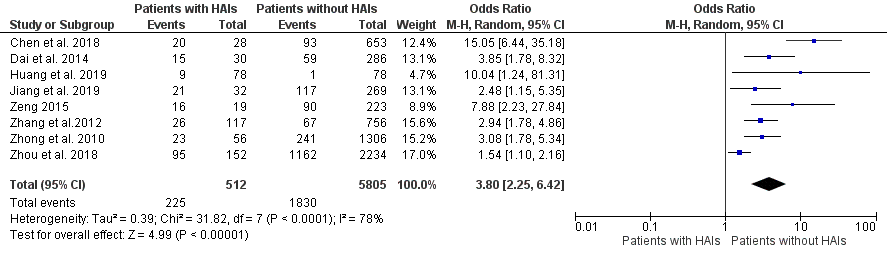

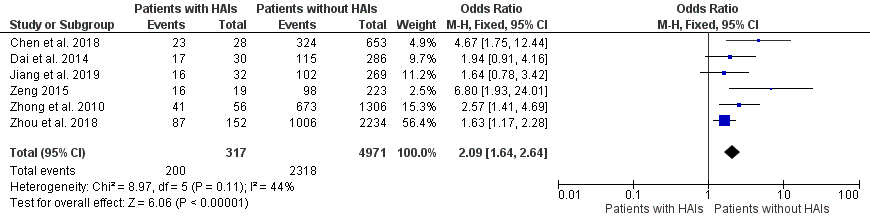
***

**Figure 1-4 Gender (Male vs. Female) Figure 1-5 Invasive procedure (Yes vs. No) Figure 1-6 Length of hospitalization (> 15 days vs. ≤ 15 days)**

**
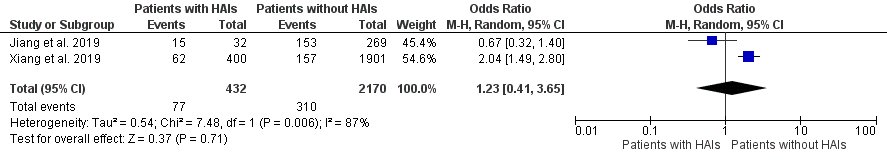

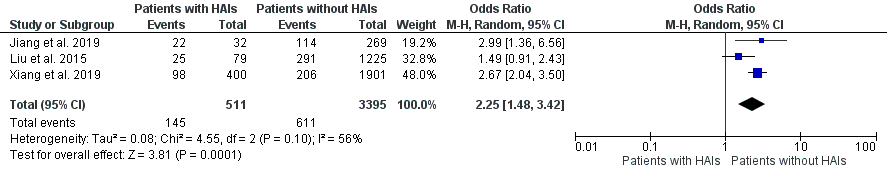

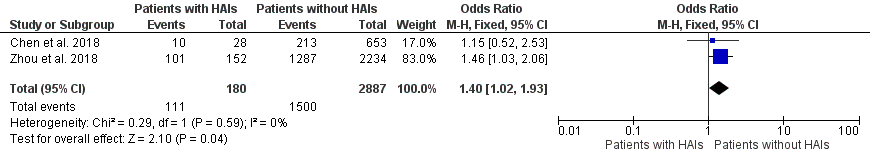
**

**Figure 1-7 Negativity of the sputum smear for acid fact staining (Yes vs. No) Figure 1-8 Secondary tuberculosis (Yes vs. No) Figure 1-9 Smoking (Yes vs. No)**

**
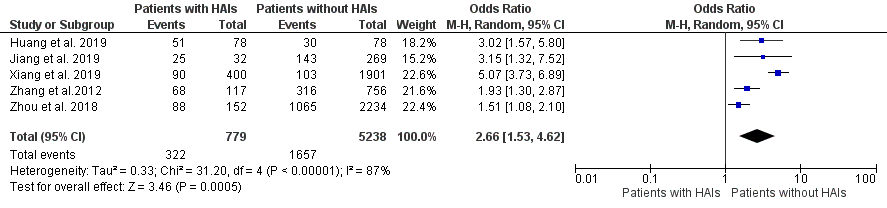

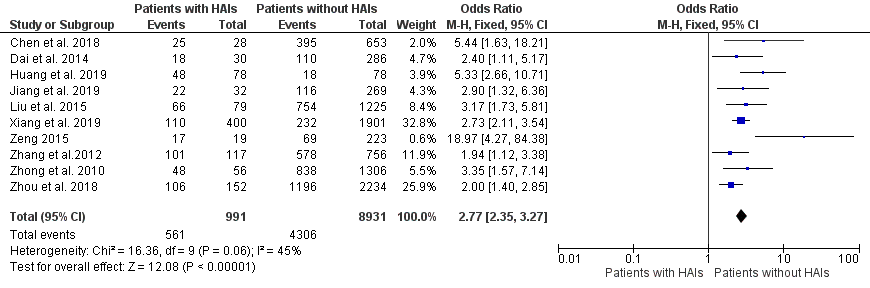
**

**Figure 1-10 Underlying disease (Yes vs. No) Figure 1-11 Use of antibiotics (Yes vs. No)**
